# The Shivplot: a graphical display for trend elucidation and exploratory analysis of microarray data

**DOI:** 10.1186/1751-0473-1-6

**Published:** 2006-11-08

**Authors:** Owen Z Woody, Robert Nadon

**Affiliations:** 1 McGill University and Genome Quebec Innovation Centre, 740 avenue du Docteur Penfield, Montreal, Quebec, H3A 1A4, Canada; 2 David R. Cheriton School of Computer Science, University of Waterloo, Waterloo, Ontario, N2L 3G1, Canada; 3 Department of Human Genetics, McGill University, Montreal, Quebec, Canada

## Abstract

**Background:**

High-throughput systems are powerful tools for the life science research community. The complexity and volume of data from these systems, however, demand special treatment. Graphical tools are needed to evaluate many aspects of the data throughout the analysis process because plots can provide quality assessments for thousands of values simultaneously. The utility of a plot, in turn, is contingent on both its interpretability and its efficiency.

**Results:**

The shivplot, a graphical technique motivated by microarrays but applicable to any replicated high-throughput data set, is described. The plot capitalizes on the strengths of three well-established plotting graphics – a boxplot, a distribution density plot, and a variability vs intensity plot – by effectively combining them into a single representation.

**Conclusion:**

The utility of the new display is illustrated with microarray data sets. The proposed graph, retaining all the information of its precursors, conserves space and minimizes redundancy, but also highlights features of the data that would be difficult to appreciate from the individual display components. We recommend the use of the shivplot both for exploratory data analysis and for the communication of experimental data in publications.

## Background

Microarrays [1] have provided a wealth of gene expression data for the biological community to interpret. The technology presents a snapshot of cellular transcription at an unprecedented level of detail, with certain array designs containing probes to assess expression levels of every known gene within the target organism's genome. Microarray data have been presented in an ever-growing number of publications, and confidence in the technology is growing as progress is made validating microarray findings.

The analysis of microarray data remains challenging, however, for a number of reasons. The technology is expensive, often restricting the number of replications that can be run in an experiment. Moreover, the validity of various statistical models and their corresponding processing algorithms for gene expression data are being actively debated [2,3].

One of the more daunting but unavoidable aspects of microarrays, as with all high-throughput systems, is the sheer volume of data that must be examined. Researchers are unable to develop comprehensive familiarity with each of the millions of data points available in a typical microarray data set. Multi-display graphical methods are thus key for reaching an understanding of the data, for assessing analysis assumptions, and for examining the effects of data pre-processing methods.

Plotting allows the rapid and concise representation of the data as a whole, and allows trends and local aberrations to be spotted with relative ease [4]. These trends are useful for more than just diagnostic assessments; due to the relative paucity of replicate observations in many studies, supplementary information is frequently gathered from observations with similar expression properties. This information can be used, for example, to obtain an improved estimate of inter-array variability [5].

A further motivation for concise and elegant graphics is the space restrictions typically imposed by scientific journals. A thorough and well designed graphic needs to be explained just once, and its subsequent re-use can allow the reader rapid insight into volumes of visual data.

The construction of comprehendible graphics, however, is a challenge of its own. Unnecessary ink must be kept to a minimum, and it is easy to obscure or even mask important points through graphs that are complex or confusing. Efficiency, readability, and relevance are of paramount concern.

With these criteria in mind, the present work illustrates an aggregate graphical approach, inspired by (but not restricted to use with) microarray data. The proposed technique pools the strengths of several other graphical methods which are commonly applied to microarray data: a boxplot, a probability density function plot, and a plot illustrating variability as a function of signal intensity. The present work demonstrates that these plots can be productively combined, facilitating integration of complementary information. Although this plot design is motivated by microarray analysis, we present this technique as a viable tool for both exploration and publication of other types of high volume multivariate data sets.

### Component plots

This section reviews the component plots that are assembled in our proposed graphical method. Simulated and empirical microarray data are used to illustrate the strengths and weaknesses of these respective plots when presented individually. The microarray data used in this section were obtained from the publicly available Affymetrix GeneChip spike-in data [6].

#### Boxplots

**Figure 1:**
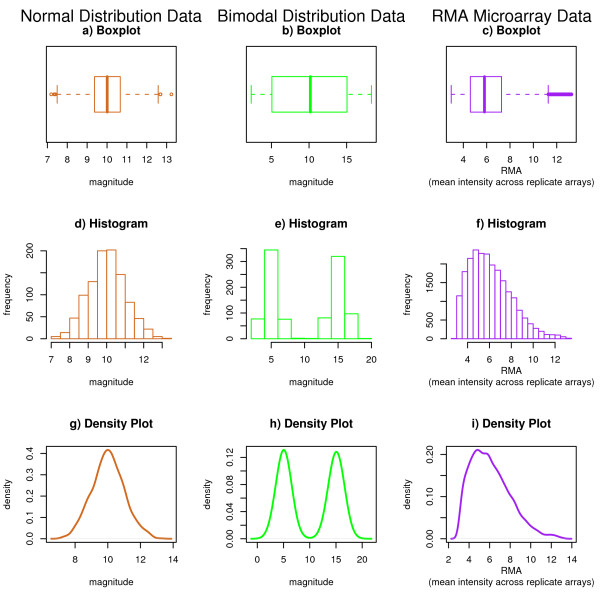
**Examples of Existing Plot Devices**. An illustration of some popular plotting techniques for distributional data. Panels a-c show horizontal boxplots; panels d-f show histograms; panels g-h show probability density plots. Plots in column 1 were constructed based on a sample of 1000 from an N(10, 1) distribution. Plots in column 2 were constructed based on a mixture; 500 drawn from N(5, 1) and 500 from N(15, 1). Column 3 applies these same plots to microarray data. The RMA algorithm was performed using 6 chips from the Affymetrix HG_U133A_tag spike-in data set; mean intensities were obtained by averaging log (base 2) summary expression measures across a subset consisting of three technical replicates.

The boxplot [7] is an example of a concise graphic in which several critical pieces of information are presented about a univariate distribution. Firstly, the median is illustrated as a dot in the "box", with the position of the dot relative to the axis giving the specific value. The interquartile range (IQR), which contains the middle 50% of the data, is shown by the length between the outer edges of the box. The points farthest from the median that are not classified as outliers are marked as the tips of the whiskers extending from the box. In addition to these five distributional landmarks, outliers are explicitly plotted as lines or dots beyond the ends of the whiskers. A rule of thumb, such as 1.5 times the IQR beyond the edges of the box, is typically employed to distinguish how far from the center an observation must be to be classified as an outlier. Examples of boxplots can be seen in Figures 1a–c.

The five-point summary delivered in the box-and-whisker portions of boxplots allows rapid access to many aspects of the distribution. For example, indications of skewness can be seen both in the position of the median within the interquartile range box (closer to either end implicates skewness) and the lengths of the respective whiskers extending from the box (if one whisker is much longer than the other, skewness is a possible explanation).

Boxplots, however, have shortcomings. Although they are easily interpreted and are pleasing to the eye, they have poor data-ink ratio [8]. The "box" component of a boxplot can be disassembled into its 5 composite values, with the remaining ink merely providing assistance to the eye. For example, only one half of the boxplot, as divided by the horizontal axis of bilateral symmetry through the center of the boxplot, is necessary.

Furthermore, enclosing a "boxed" region encourages the interpretation of area, a quantity which is arbitrarily determined by the boxplot's vertical width. This two-dimensional depiction only distracts from the interpretation of one-dimensional distance. Although the marked quantities and their positions relative to one another are relevant, a majority of the lines surrounding and joining these values are not. Boxplots also potentially mask underlying features of the distribution, such as tail densities and multiple modes [9]. As an illustration of this hazard, compare the boxplot generated from a normal distribution (Figure 1a) to the boxplot of a distinctly bimodal (mixture of two non-overlapping normals) distribution (Figure 1b). From these two illustrations alone, it would be very difficult to discern that the two distributions have a different number of modes. This information can be recovered, however, through use of the density plot.

#### Density plots

Perhaps the easiest way to introduce the density plot is to first examine the related concept of a histogram [10]. Histograms work on a univariate data set by first stratifying the data into "bins" based on value, and then plotting the population count of each bin. The resulting graph displays how the data values are distributed; bins containing many observations have taller bars than sparsely populated bins. Histograms are excellent for detecting multiple modes, skewness, and kurtosis. Examples of histograms can be seen in Figures 1d–f.

Density plots [11] offer what is in essence a "smoothed" histogram; instead of using discrete bins and counts, density plots employ a continuous curve to communicate the same information. Area is used to convey the probability of observations within specified ranges. Specifically, were a sample value drawn from the depicted distribution, the probability of this value lying within a given interval can be determined by taking the integral of the curve bounded by that interval. Example density plots can be seen in Figures 1g–i. Like the histogram, density plots are excellent for detecting multiple modes, skewness, and kurtosis. The limitation of any approach that employs frequency as a metric is that extreme values and outliers are given little credence. These outlying values can carry a high leverage on important quantities such as the mean, yet they have only a subtle effect on the density curve. Although density plots do an excellent job estimating the position and number of modes, the mode is itself a highly variable estimator of the center of a distribution, and it is challenging to visually estimate the position of a mean or median from the density curve.

#### Variability-versus-intensity plots

It is typical for the variability of replicated microarray data to exhibit a dependence on intensity, whether replication is across or within arrays and whether the variability reflects processing effects only or processing plus biological effects. One common approach is to log gene expression data, which tends to stabilize error variance across replicates for mid-to-upper range intensity values but which has the disadvantage of inflating additive error for low intensity values. Alternative approaches to microarray data transformation model both additive and proportional error components (see the "generalized log" methods of [12-16]). Variability versus intensity plots can be used to assess how successfully such transformations have stabilized the variance throughout the entire intensity range. Additionally, the plot can also be used as a visual aid for pooling estimates of random error associated with genes of similar expression intensity [3,17,18].

**Figure 2:**
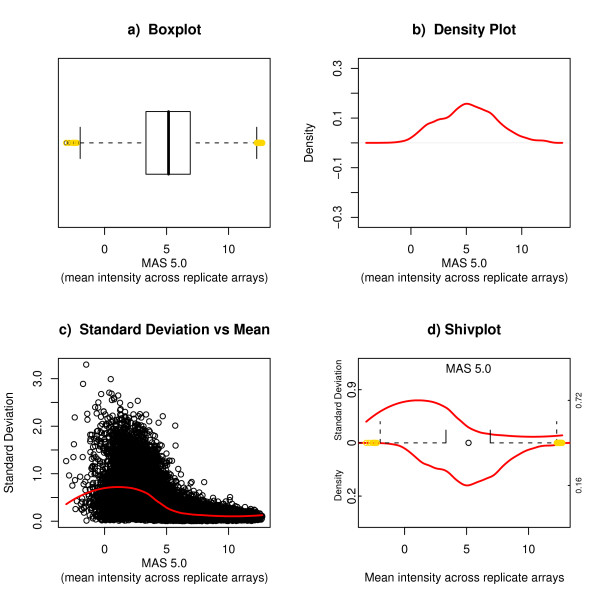
**The Shivplot and its Components**. Illustration of the individual components of a shivplot are displayed in a-c; the agglomeration of these plots is displayed in the shivplot in d. The same raw data as in Figure 1 was instead processed with the Affymetrix MAS 5.0 default algorithm (as implemented in Bioconductor [25]), and the resulting expression measures are illustrated here. Means of log (base 2) measurements were taken across technical replicates.

The relationship of variability to mean intensity (i.e. the trend line) is the important piece of information to be obtained from this plot. The vast majority of points on the plot are of little interest to the viewer and can make the visual estimation of the trend challenging when data points are densely packed. As a method of capturing the trend of variability as a function of mean intensity, one can employ any of the loess family of smoothers [4,19], which perform local weighted least squares regression to capture nonlinear trends in data. (An example loess fit can be seen in Figure 2c.)

## Results and discussion

### The Shivplot

As can be seen from Figure 2a–c, all three of the discussed plots – boxplot, density plot, and variability-versus-intensity (standard deviation vs mean) – have an axis in common. Figure 2d presents the shivplot, an amalgamation of the three plots. To first capture the essence of the boxplot, we eliminate its vertical width, thereby reducing it down to point components: the five distributional landmarks along with the outliers. These points can be displayed as ticks across the middle of the graph, at y = 0. Then, in the region below, we can draw the reflection of the density function plot (with the scale starting at zero and increasing downward along the lower half of the y-axis). The area enclosed by this curve below y = 0 represents the probability density. Lastly, we can draw the loess fit from the variability-versus-intensity plot in the upper half of the graph. One can quickly isolate the information in any of the three precursor graphs from the final image – there is no significant loss of information when the plots are superimposed. (The values along the right side of the graph provide maximum values: the maximum standard deviation above, and the maximum density below.)

The data interpreter needs to adjust to two aspects of this graph: The upper and lower halves of the plot have distinct y-axes, with different scales and units, and the density distribution is inverted. However, having the three graphs simultaneously available provides the advantage that each plot can readily be interpreted in the context of the other two. The advantages of this feature are best illustrated by examining empirical data, as provided in the following section.

### Examples

There is an ongoing debate about the appropriate analysis of data derived from microarrays. As such, it is of great interest to biologists and statisticians alike to observe the impact of different statistical algorithms on microarray data. Having approachable visual tools with which to make such comparisons greatly expedites this tedious and iterative process.

The examples in this section come from three different data sets. To build familiarity with the shivplot, we use data from the Affymetrix U133 spike-in data set to demonstrate the appearance of the components of the shivplot, and then follow this up with their shivplot equivalent. From these shivplots, we will be able to evaluate distributional features that distinguish the expression estimates produced by different Affymetrix normalization procedures from one another.

Beyond distinguishing the nuances of algorithms, the shivplot can also be used to detect interesting distributional properties of data sets that typical exploratory graphics may understate or miss. To demonstrate this utility, we have obtained the data from two microarray studies that were specifically designed to examine technical aspects of microarray data structure. Specifically, data sets produced by [20] and [21] will be examined, as these data sets highlight unique advantages of the shivplot in an exploratory context.

**Figure 8:**
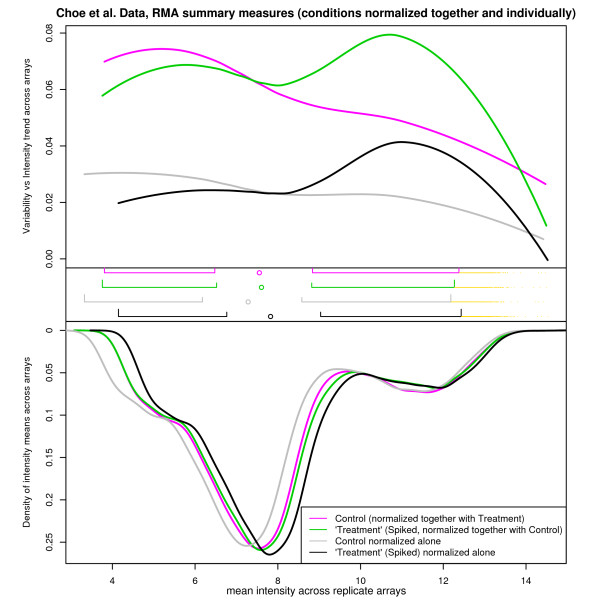
**A multi-sample Shivplot on the Choe data following RMA normalization**. Two interpretations of each of the conditions of the Golden Spike are illustrated here. The "treatment" and "control" data were generated by normalizing both conditions (6 chips) simultaneously, and then plotting each condition individually. To produce the "control alone" and "treatment alone" lines, RMA was performed on each of the conditions individually (3 chips). Although this is less than the recommended minimum for an RMA procedure, we were interested to see whether the different variance vs intensity relationships were produced as an artifact during normalization. This does not appear to be the case.

**Figure 9:**
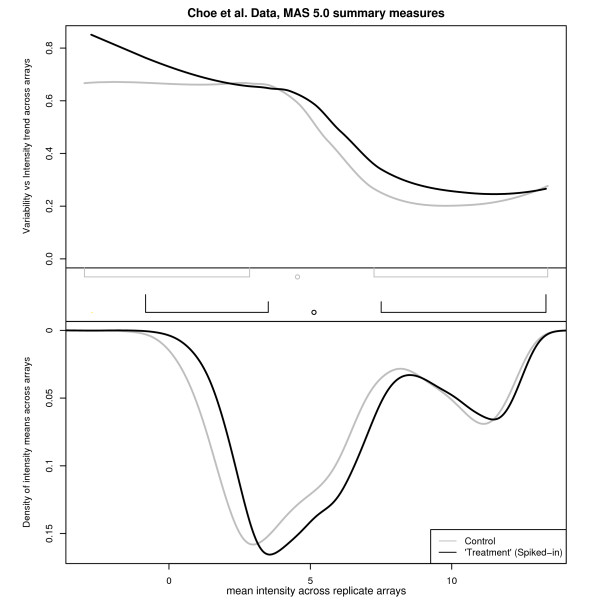
**A multi-sample Shivplot on the Choe data following MAS 5.0 normalization**. This figure contains MAS 5.0 normalizations of the same data depicted in Figure 8. Note that since MAS 5.0 does not use information across arrays, there is no distinction between normalizing arrays together or separately. Note that the variance vs intensity relationship is common across conditions following MAS 5.0 normalization, except for a small number of low intensity measurements, in contrast to Figure 8's RMA normalization of the data.

For these latter two data sets, we also present a slight variation on our shivplot theme. When a large number of distributions are to be compared in tandem, granting each shivplot its own unique frame and axes may require too much space to be efficient. Separate frames also make it difficult to perceive inter-relationships among density, variability, and signal intensity. To address these issues, we introduce a multi-sample shivplot (Figures 8 and 9), in which each distribution is depicted upon a common set of axes.

#### Affymetrix spike-in data

As mentioned earlier, the microarray manufacturing company Affymetrix has produced spike-in data sets where the underlying transcript quantity information is engineered and thus known in advance. Such data sets have been used to design [22], field test [23], and compare [24] statistical algorithms. The specific details of these spike-in experiments can be found at the aforementioned website. We shall use our shivplot to compare a subset of Affymetrix analysis algorithms.

**Figure 3:**
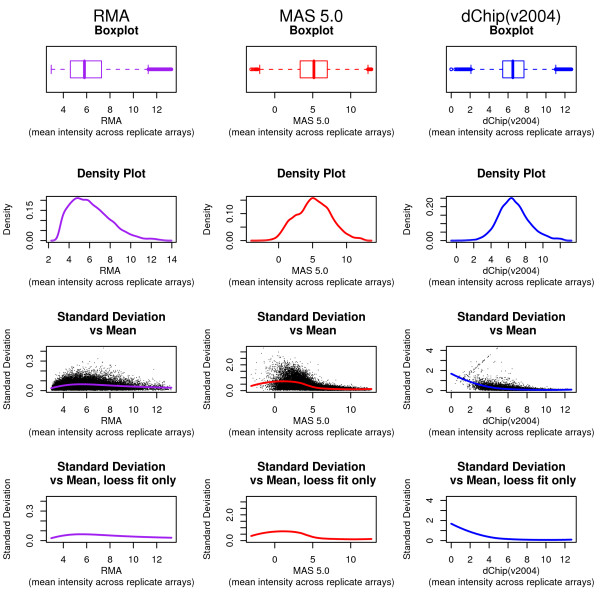
**Algorithm Comparison with Multiple Plots**. An illustration of popular plots on microarray data. Three high-impact microarray analysis algorithms are compared side-by-side; column 1 features expression measures produced by the RMA algorithm (as in Figure 1), column 2 contains expression measures produced by MAS 5.0 (as in Figure 2), and column 3 contains data preprocessed with the dChip algorithm (software version '2004'). The same data set as in Figures 1 and 2 was passed into each algorithm, and the resulting means across a fixed set of three technical replicates are compared in these plots.

**Figure 4:**
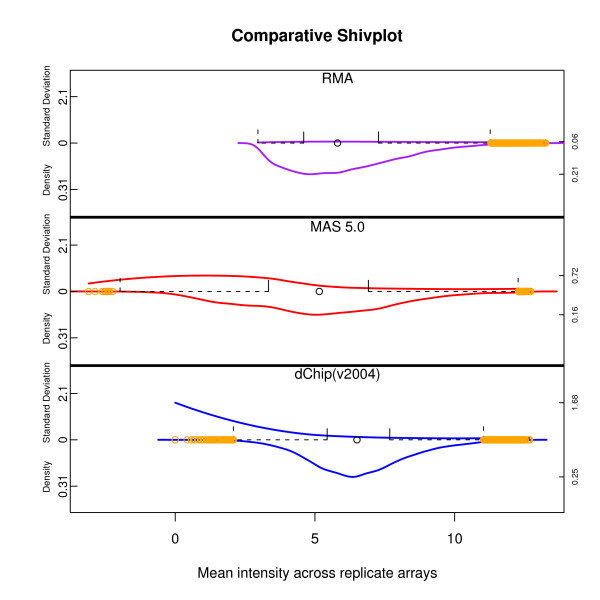
**Algorithm Comparison using Shivplot**. The same data as in Figure 3, in shivplot form. Note that the upper (variability) y-axis and lower (density) y-axis share a scale across the three plots. This allows us to readily observe, for example, how much smaller the variability across replicates is for RMA compared to either MAS 5.0 or dChip.

Figure 3 shows the individual components of the shivplot produced by three algorithms: MAS 5.0 [22], as implemented in [25], RMA [23], as implemented in [25], and dChip [26], as implemented in their dChip software, version '2004'. Figure 4 shows the shivplot representation of these data. Note that a wide variety of information can be gleaned from this side-by-side shivplot comparison. For example, the three metrics clearly do not share an absolute scale. Furthermore, they are in disagreement over the general distribution of points. RMA, for example, has a larger proportion of very small values compressed within a small range and with outliers solely in the high expression range compared to the other examined algorithms. Both MAS 5.0 and dChip, by contrast, produce approximately symmetrical distributions, as can be seen in the density portion of the shivplot.

MAS 5.0 has a step in its algorithm in which unrealistically small gene expression values are replaced with imputed values. The consequences of this approach can be seen uniquely in the MAS 5.0 shivplot as a decrease in variability across replicates at the (partially imputed) low end. A similar dip in average variability can be see at the low end of the RMA intensity spectrum, although this phenomenon does not have a satisfying methodological explanation like the one offered for MAS 5.0. The comparative plot also shows the substantially lower replicate variability of RMA relative to MAS 5.0 and dChip, a feature of the RMA algorithm that has been well documented [24]. Though dChip has relatively stable variance at the high end, there is an escalation in variability near the low end.

**Figure 5:**
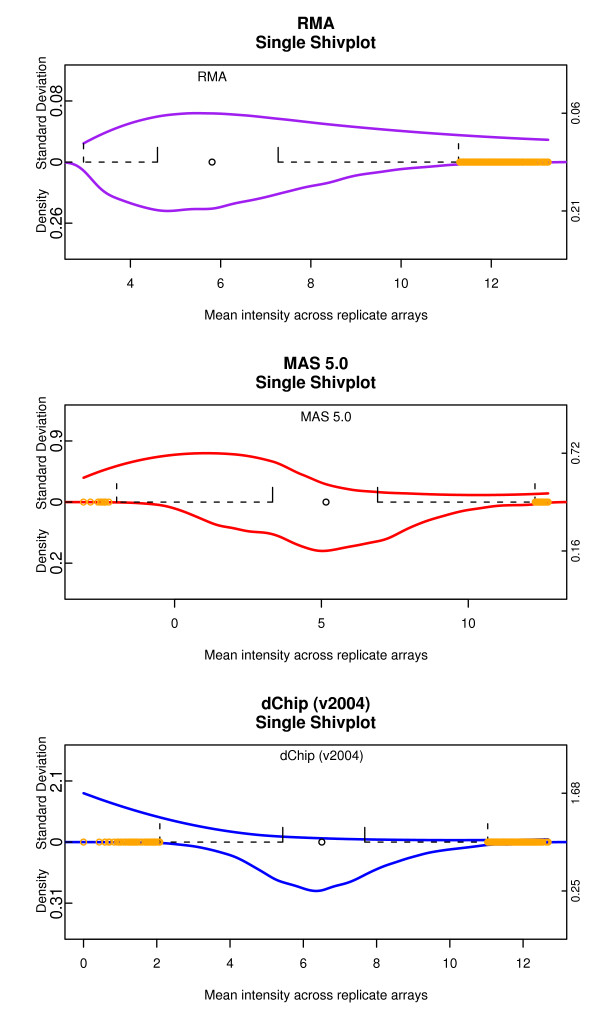
**An Alternate Construction of Shivplot**. The same shivplots as in Figure 4 with each axis scaled individually. This local scaling makes it easier to notice features, such as the dependence of RMA variability across replicates on the mean, that were out of resolution when a common scale was enforced.

Figures 5a–c illustrate the shivplots scaled individually. Note that on this plot it is much easier to detect details such as the non-uniformity (relative to intensity) of RMA cross-replicate variability, a detail which was lost when the RMA variability was scaled against the comparatively massive variability of the dChip low end.

#### Kendziorski et al. pooling data

**Figure 6:**
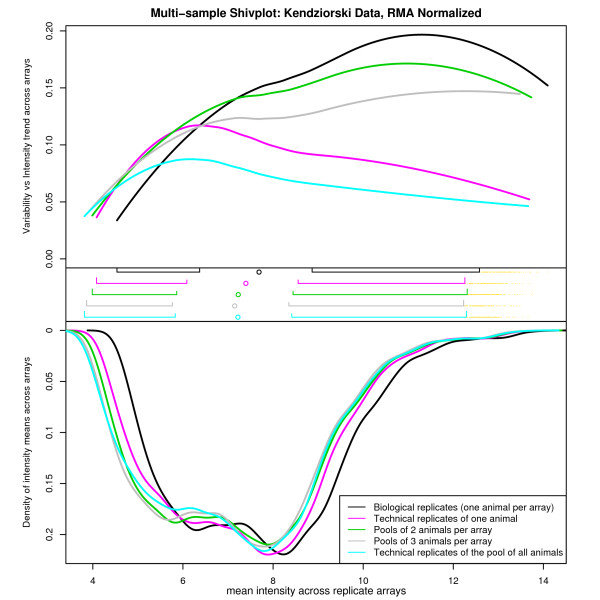
**A multi-sample Shivplot on RMA'd Data from the Pooling Experiment**. To produce this figure, RMA normalization was applied to each level of replication separately, producing one data set per such level. Each of these data sets is represented by its own line on the common set of shivplot axes. The most striking feature of this illustration is the clear distinction between conditions which include some measure of biological variance (pools of one, two, or three animals) and those in which the inter-array variability is purely technical (pooling all animals, or technical replicates of a single animal).

This study, assessing the merits of pooling biological samples, examined whether array-to-array variability was directly related to degree of pooling (i.e., 2 or 3 samples pooled per hybridization) as would be predicted by theory. Additionally, several technical array replications were performed on a subset of the samples used in the study, allowing for an evaluation of array-to-array variability when biological material is invariant. Five types of replication were taken from this study – biological replication, in which one animal is used per array, pools of 2 (or 3) animals, where multiple animal samples are pooled on each array, and two levels of technical replication – one where the sample taken from a single animal is processed on multiple arrays, and one where a pool of all available animal samples is processed on multiple arrays. Note that in the latter two groups, the array-to-array variability is purely technical – the same sample material is applied to each array. The former three capture various levels of "biological" variability – that is to say, an estimate of how much gene expression varies from animal to animal when treatment is invariate. Note that one would expect the observed inter-array variability to diminish as more animals are pooled per array (as pooling should act as a sort of "biological averaging" prior to hybridization). A multi-sample shivplot can be used to examine relationships and trends in the variability of feature expression levels as a function of both degree of pooling and/or normalization strategy. Figure 6 depicts the variability vs intensity relationship when the RMA normalization procedure is used to produce expression summary measures for each level of pooling individually. As shown by the top part of the shivplot (the variability vs intensity component), there is a striking difference at the high end between groups that retain some degree of biological variability (biological replicates, as well as pools of 2 and 3 animals) and those within which variability is purely technical (replicates of the same animal or replicates of the pool of all animals). Furthermore, for the medium-to-high end, the magnitude of the variability corresponds nicely with the amount of pooling, with pools of fewer animals producing more variable expression estimates than those with several animals.

**Figure 7:**
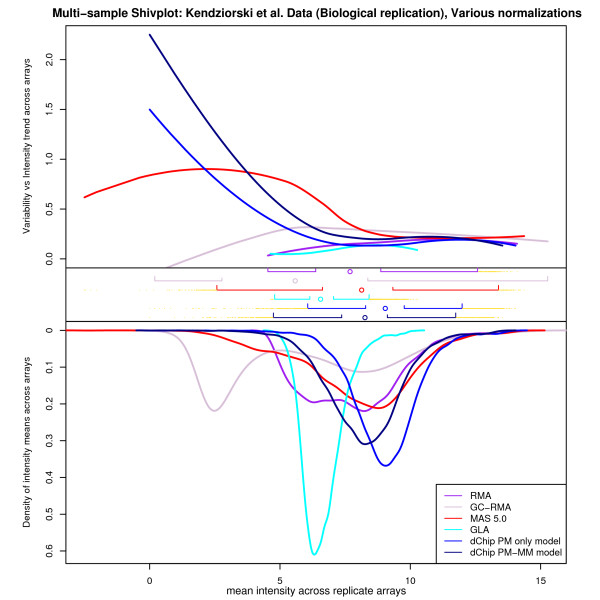
**A multi-sample Shivplot With Many Normalizations on a Single Condition of the Pooling Experiment**. To produce this figure a single condition from the Kendziorski data (single animals per chip) was normalized using a number of alternative Affymetrix summarization algorithms.

Note that the simultaneous interpretation offered by the multi-sample shivplot is particularly helpful in making assessments about observed trends. Had only boxplots and/or expression density plots been examined, the distinction between data sets based on variability would have been invisible. On the other hand, however, solely examining the variability plot would allow the viewer to notice these trends but not allow any evaluation of their consequence. Differences at the low and high ends of measurement spectra may simply reflect limited sample size, and it is thus crucial to be able to supplement any difference in variability trend with information about the relative number of observations affected. If the differences only concern the lowest 5% of the data, for example, any trends observed may be artifacts of sampling, and even real effects may have little consequence when so few data points are influenced. Moreover, note that despite differences in the variance-intensity relationship, the distributions of the intensity measurements were highly similar. This information is difficult to recover when examining the components individually. Figure 7 was generated by examining various normalization strategies on biological replicates of a common experimental condition (with no pooling). Two additional normalization procedures are introduced in this figure, the GC-RMA [27] and GLA [28] algorithms. This allows us to observe how the various normalization algorithms influence trend in the variability observed in Figure 6, and how they affect the distribution of the data. Note that in this shivplot, the importance of the simultaneous presentation of the components is again evident. In particular, the variability plot alone would tempt the interpretation that the normalization algorithms can be roughly divided into two groups – those that have elevated low-end variability and those that do not. However, the boxplot and distribution density plots reveal that the story is more complicated. Specifically, although the measures with high variance at the low end seem to have relatively homogenous density distributions, the measures with low variance at the low end have strikingly different density distributions, resulting in completely different interpretations of the distribution of the same biological data (i.e. GC-RMA is bimodal, RMA is platykurtotic and possibly also bimodal, GLA has compressed range). This provides another example where examining the components of the shivplot individually would reduce their interpretability.

#### Choe et al. Golden Spike data

The final data set we examine here was the Golden Spike experiment produced for [21]. This data set was engineered so that a vast majority of the effect sizes between treatment and control condition were known in advance, as determined by a predetermined spike-in design. Although designed to serve as a benchmarking data set for algorithm comparison, the golden spike data set produced results at odds with other benchmark data sets and has been critiqued on methodological grounds [29]. More specifically, it was remarkable that in the comparison performed in [21], the standard RMA algorithm performed quite poorly, and in particular worse than MAS 5.0. This finding contrasts with results of other method comparison studies. In an attempt to identify a possible explanation for this unusual result, we apply the shivplot successively to the RMA and the MAS 5.0 processing of the Golden Spike expression data.

On one hand, Figure 8 presents a multi-sample shivplot of RMA normalized data, distinguished by group and by whether normalization was performed across conditions. It is noteworthy that the variance vs intensity relationship is quite different across conditions, even after executing the very strong quantile normalization procedure, integral to RMA, which largely homogenizes the expression measure distributions. This effect persists regardless of whether the conditions are normalized together or separately. As such, quantile normalization did not produce the variability discrepancy. An explanation of this discrepancy would be of relevance to anyone using variability to weight the significance of differential expression.

As in the analysis of the previous data set, the shivplot's simultaneous presentation of the variability-vs-intensity plot and the density distribution is clearly advantageous. Had the variability plot been examined individually, it would have been challenging to correctly predict the homogeneity of the component distributions. Similarly, it would have been difficult to predict from the highly similar distributional plots that the variability relationship would differ so markedly. The findings in this plot offer a graphical explanation why the (unusual) second round of normalization was deemed necessary by [21] to analyze their data.

Figure 9, on the other hand, which presents the multi-sample shivplot of the MAS 5.0 normalized data, tells a different story. In contrast to the crossing trend lines observed in Figure 8, the variance-intensity relationships for the control and treatment groups were highly similar, except for a small number of low intensity measurements.

It seems likely that either RMA has produced this unusual difference between groups (although, as we have demonstrated, the quantile normalization is unlikely to blame), or some aspect of the MAS 5.0 normalization procedure has managed to eliminate it from the raw data. While further detective work is beyond the scope of the present work, the shivplot was a pivotal tool in uncovering this intriguing phenomenon in the Golden Spike data.

### Availability

**R/S-PLUS **code written by the authors to produce shivplots is available in a flat file [see Additional File 1]. Also provided are a tutorial demonstrating the use of the code [see Additional File 2] and example data sets formatted for use with the code [see Additional Files 3, 4]. Alternatively, the entire shivplot library is available as a package for **R **in either source form [see Additional File 5] or as a Windows binary [see Additional File 6].

## Conclusion

Microarray data, like data obtained from all high-throughput assays, represent a complex system, and significant amounts of space must be allocated in each publication to introduce and familiarize the reader with the technology and methodology used in any analyses. With the space restrictions imposed by many scientific journals, it is important to present as much information as cleanly as possible in concise graphics. The present work illustrates how three graphical tools of growing importance to microarrays and other high-throughput platforms can be effectively combined into a single plot, not only saving space but facilitating insights obtainable from complementary simultaneous interpretation. Furthermore, the introduced tool is general enough to serve a variety of high-throughput purposes, both in microarray data analysis and exploratory data analysis in general.

## Supplementary Material

Additional file 1 **shivcode.txt**. This file contains shivplot script code for both **R **and **S-PLUS**. To prepare an **R/S-PLUS **session to produce shivplots, either copy the contents of this file into the input console of an instance of **R/S-PLUS **or read the code in using the source() command (see tutorial.txt). There are several prepackaged plots included as examples. The instructions to produce these plots can be reviewed at any time by calling the shivHelp command. Click here for file

Additional file 2 **tutorial.txt**. This file provides instructions on how to read custom data into **R **or **S-PLUS**, and provides some examples of how to call the shivplot function. This is a good place to start for those unfamiliar with **R **or **S-PLUS**. Click here for file

Additional file 3 **example.txt**. This file contains some artificial data in a flat file format that is easily manipulated by **R **and **S-PLUS**. It is used in the tutorial, and can be used as a file format template for those unfamiliar with the import utilities of **R **and **S-PLUS**. Click here for file

Additional file 4 **alternate.txt**. This file contains some artificial data in a flat file format that is easily manipulated by **R **and **S-PLUS**. It is used in the tutorial, and can be used as a file format template for those unfamiliar with the import utilities of **R **and **S-PLUS**. Click here for file

Additional file 5 **source distribution of R shivplot package**. This file is a compressed archive of the source files necessary to build the shivplot library in R. Click here for file

Additional file 6 **Windows binary distribution of R shivplot package**. This file is a compressed archive of the windows binary distribution of the shivplot library. Click here for file
