# Supplementary material for: The Shivplot: a graphical display for trend elucidation and exploratory analysis of microarray data
Source: Source Code Biol Med. 2006 Nov 8;1:6. doi: 10.1186/1751-0473-1-6 (PMC1679802; doi:10.1186/1751-0473-1-6)
Supplement: Additional file 6 — Windows binary distribution of R shivplot package. This file is a compressed archive of the windows binary distribution of the shivplot library. [file 1751-0473-1-6-S6.addi › shivplots_1.0.zip!/shivplots/html/00Index.html]

R: Shivplot plotting utility.

# Shivplot plotting utility.

---

## Documentation for package `shivplots' version 1.0

## Help Pages

|  |  |
| --- | --- |
| shivplots-package | This package contains functions to create shivplots. |
| densityPlugin | Density Plug-in for Shivplot |
| exportAll | Produce Figures 1-5 from the Shivplot Manuscript |
| multishivplot | The Multi-Sample Shivplot |
| sdVsLocPlugin | Variability vs Location Plug-in for Shivplot |
| shivplot | The Shivplot: a graphical display for trend elucidation and exploratory analysis of microarray data |
| shivplots | This package contains functions to create shivplots. |
| shivSample | Shivplot Example |
