# Supplementary material for: The Shivplot: a graphical display for trend elucidation and exploratory analysis of microarray data
Source: Source Code Biol Med. 2006 Nov 8;1:6. doi: 10.1186/1751-0473-1-6 (PMC1679802; doi:10.1186/1751-0473-1-6)
Supplement: Additional file 6 — Windows binary distribution of R shivplot package. This file is a compressed archive of the windows binary distribution of the shivplot library. [file 1751-0473-1-6-S6.addi › shivplots_1.0.zip!/shivplots/html/addslash.html]

R: Add Slashes to Path

|  |  |
| --- | --- |
| addslash {shivplots} | R Documentation |

## Add Slashes to Path

### Description

Given a string representation of a path, append a terminal slash if one is not present.
For compatibility with shivplot output operations.

### Usage

```
addslash(word)
```

### Arguments

|  |  |
| --- | --- |
| `word` | a character string, typically a file path (e.g. an output directory) |

### Value

Returns the input string, with an appended terminal "//" if one was
not present on the input.

### Author(s)

Owen Woody <owoody@uwaterloo.ca>

### Examples

```
#These should produce the same output:
test1 = addslash("D://test//")
test2 = addslash("D://test")
print (test1); print(test2); print(paste("Equivalent?:", test1==test2));
```

---

[Package *shivplots* version 1.0 Index]
