# Supplementary material for: The Shivplot: a graphical display for trend elucidation and exploratory analysis of microarray data
Source: Source Code Biol Med. 2006 Nov 8;1:6. doi: 10.1186/1751-0473-1-6 (PMC1679802; doi:10.1186/1751-0473-1-6)
Supplement: Additional file 6 — Windows binary distribution of R shivplot package. This file is a compressed archive of the windows binary distribution of the shivplot library. [file 1751-0473-1-6-S6.addi › shivplots_1.0.zip!/shivplots/html/densityPlugin.html]

R: Density Plug-in for Shivplot

|  |  |
| --- | --- |
| densityPlugin {shivplots} | R Documentation |

## Density Plug-in for Shivplot

### Description

This is a function to generate a density curve, processing the data
in a form where they can be used as one of the two components (top or bottom)
of a shivplot.

### Usage

```
densityPlugin(dataxx, LOCATION.FCN)
```

### Arguments

|  |  |
| --- | --- |
| `dataxx` | a matrix of input data: one row per variable, one column per replication |
| `LOCATION.FCN` | the location function being used to find a centre across replicates (i.e. mean, median). Mean is passed by default from shivplot() |

### Details

The location function is applied to rows of the input matrix to produce one summary measure per row.
A density curve is then fit to model the distribution of these summaries.

### Value

A list:

|  |  |
| --- | --- |
| `x` | x coordinates of points along the estimated density curve. |
| `y` | y coordinates of points along the estimated density curve. |
| `ylab` | short y-axis label for use when space is limited. |
| `ylablong` | long y-axis label for when space is not an issue. |

### Note

This function can be used as a template for alternative plug-in components of the shivplot display.

### Author(s)

Owen Woody, <owoody@uwaterloo.ca>

### References

Woody, O.Z. and Nadon, R. (2006) *The Shivplot: a graphical display for trend elucidation and exploratory analysis of microarray data.* To appear in Source Code for Biology and Medicine, www.scfbm.org

### See Also

`shivplot`, `multishivplot`

---

[Package *shivplots* version 1.0 Index]
