# Supplementary material for: The Shivplot: a graphical display for trend elucidation and exploratory analysis of microarray data
Source: Source Code Biol Med. 2006 Nov 8;1:6. doi: 10.1186/1751-0473-1-6 (PMC1679802; doi:10.1186/1751-0473-1-6)
Supplement: Additional file 6 — Windows binary distribution of R shivplot package. This file is a compressed archive of the windows binary distribution of the shivplot library. [file 1751-0473-1-6-S6.addi › shivplots_1.0.zip!/shivplots/html/exportAll.html]

R: Produce Figures 1-5 from the Shivplot Manuscript

|  |  |
| --- | --- |
| exportAll {shivplots} | R Documentation |

## Produce Figures 1-5 from the Shivplot Manuscript

### Description

Creates Postscript versions of figures 1-5 from the shivplot manuscript.
If the data used in the manuscript are not readily available, random data are used instead.

### Usage

```
exportAll(outputD = "D://", actualData = FALSE)
```

### Arguments

|  |  |
| --- | --- |
| `outputD` | path to a location where (postscript) output should be created |
| `actualData` | flag: Are the data used to produce the manuscript available in the output directory? |

### Author(s)

Owen Woody, <owoody@uwaterloo.ca>

### References

Woody, O.Z. and Nadon, R. (2006) *The Shivplot: a graphical display for trend elucidation and exploratory analysis of microarray data.* To appear in Source Code for Biology and Medicine, www.scfbm.org

### See Also

`shivplot`, `multishivplot`

### Examples

```
##Not run:
##Produce Figures 1-5 as postscripts (using random data) and put output in D://temp//
## Not run: exportAll("D://temp//")
```

---

[Package *shivplots* version 1.0 Index]
