# Supplementary material for: The Shivplot: a graphical display for trend elucidation and exploratory analysis of microarray data
Source: Source Code Biol Med. 2006 Nov 8;1:6. doi: 10.1186/1751-0473-1-6 (PMC1679802; doi:10.1186/1751-0473-1-6)
Supplement: Additional file 6 — Windows binary distribution of R shivplot package. This file is a compressed archive of the windows binary distribution of the shivplot library. [file 1751-0473-1-6-S6.addi › shivplots_1.0.zip!/shivplots/html/multishivplot.html]

R: The Multi-Sample Shivplot

|  |  |
| --- | --- |
| multishivplot {shivplots} | R Documentation |

## The Multi-Sample Shivplot

### Description

An alternative presentation of multiple shivplots where multiple lines (one per data set) are simultaneously displayed on a common plot surface.

### Usage

```
multishivplot(data, plotColors, lineTypes, plotNames, titles = "", FUN1 = sdVsLocPlugin, FUN2 = densityPlugin, locationFCN = mean, boxWidth = 0.3, whiskWidth = 0.5, outlierColor = "gold", outlierCharacter = -1, xLab = "mean intensity across replicate arrays")
```

### Arguments

|  |  |
| --- | --- |
| `data` | a list whose entries are matrices with rows representing subjects and columns representing observations |
| `plotColors` | what colors to use for each created plot |
| `lineTypes` | what line types to use for each created plot |
| `plotNames` | names to attribute to each individual shivplot |
| `titles` | the title for the set of plots |
| `FUN1` | the function to use to compute the line on the top half of the shivplot. By default, this function relates standard deviations across replicates to the selected measure of centre |
| `FUN2` | the function to use to compute the line on the bottom half of the shivplot. By default, this function estimates the density curve of the distribution of measures of centre |
| `locationFCN` | the function to use to obtain an estimate of location across replicate observations. The default measure (and the one used in the manuscript) is the mean |
| `boxWidth` | controls the length of the ticks drawn to denote the 'box' region along the x-axis |
| `whiskWidth` | controls the length of the ticks drawn to denote the whiskers along the x-axis |
| `outlierColor` | the color to use to mark observations that qualify as outliers |
| `outlierCharacter` | the plotting character to use to mark outliers |
| `xLab` | the label for the shared x-axis |

### Author(s)

Owen Woody, <owoody@uwaterloo.ca>

### References

Woody, O.Z. and Nadon, R. (2006) *The Shivplot: a graphical display for trend elucidation and exploratory analysis of microarray data.* To appear in Source Code for Biology and Medicine, www.scfbm.org

### See Also

`shivplot`

### Examples

```
##not run:
#Generate a multishivplot on some nonsense data
## Not run: 
data <- list (a = matrix(rnorm(300, 10, 1), ncol=3), b = matrix(runif(300, 8, 12), ncol=3))
multishivplot(data, plotNames = c("normal", "unif"), titles="example")
## End(Not run)
```

---

[Package *shivplots* version 1.0 Index]
