# Supplementary material for: The Shivplot: a graphical display for trend elucidation and exploratory analysis of microarray data
Source: Source Code Biol Med. 2006 Nov 8;1:6. doi: 10.1186/1751-0473-1-6 (PMC1679802; doi:10.1186/1751-0473-1-6)
Supplement: Additional file 6 — Windows binary distribution of R shivplot package. This file is a compressed archive of the windows binary distribution of the shivplot library. [file 1751-0473-1-6-S6.addi › shivplots_1.0.zip!/shivplots/html/sdVsLocPlugin.html]

R: Variability vs Location Plug-in for Shivplot

|  |  |
| --- | --- |
| sdVsLocPlugin {shivplots} | R Documentation |

## Variability vs Location Plug-in for Shivplot

### Description

This is a function to generate a lowess curve fit modelling variability as a function of "intensity", processing the data
in a form where they can be used as one of the two components (top or bottom)
of a shivplot.

### Usage

```
sdVsLocPlugin(dataxx, LOCATION.FCN)
```

### Arguments

|  |  |
| --- | --- |
| `dataxx` | a matrix of input data: one row per variable, one column per replication |
| `LOCATION.FCN` | the location function being used to find a centre across replicates (i.e. mean, median). Mean is passed by default from shivplot() |

### Details

The location function is applied to rows of the input matrix to produce one summary measure per row.
Similarly, the variability (as standard deviation) is calculated for each row of the input matrix.
A lowess curve is then fit to the scatterplot of variability vs location, and points tracking the resulting curve are returned.

### Value

A list:

|  |  |
| --- | --- |
| `x` | x coordinates of points along the fitted lowess curve. |
| `y` | y coordinates of points along the fitted lowess curve. |
| `ylab` | short y-axis label for use when space is limited. |
| `ylablong` | long y-axis label for when space is not an issue. |

### Note

This function can be used as a template for alternative plug-in components of the shivplot display.

### Author(s)

Owen Woody, <owoody@uwaterloo.ca>

### References

Woody, O.Z. and Nadon, R. (2006) *The Shivplot: a graphical display for trend elucidation and exploratory analysis of microarray data.* To appear in Source Code for Biology and Medicine, www.scfbm.org

### See Also

`shivplot`, `multishivplot`

---

[Package *shivplots* version 1.0 Index]
