# Supplementary material for: The Shivplot: a graphical display for trend elucidation and exploratory analysis of microarray data
Source: Source Code Biol Med. 2006 Nov 8;1:6. doi: 10.1186/1751-0473-1-6 (PMC1679802; doi:10.1186/1751-0473-1-6)
Supplement: Additional file 6 — Windows binary distribution of R shivplot package. This file is a compressed archive of the windows binary distribution of the shivplot library. [file 1751-0473-1-6-S6.addi › shivplots_1.0.zip!/shivplots/html/shiv.determineGlobals.html]

R: Determine Axis Limits Which Accomodate Data to be Plotted

|  |  |
| --- | --- |
| shiv.determineGlobals {shivplots} | R Documentation |

## Determine Axis Limits Which Accomodate Data to be Plotted

### Description

Determine common axis limits which accomodate each data set to be plotted.

### Usage

```
shiv.determineGlobals(numPlots, plotData)
```

### Arguments

|  |  |
| --- | --- |
| `numPlots` | the number of shivplots to be constructed |
| `plotData` | the corresponding data for each shivplot to be constructed |

### Details

This is an internal function of 'shivplot' - it was not meant to be called directly.

### Value

A list:

|  |  |
| --- | --- |
| `maxLoc` | largest location value (upper x limit) |
| `minLoc` | smallest location value (lower x limit) |
| `centreLoc` | an estimate of the "centre" of the plotting area, for label location |
| `maxTop` | the largest y-value for the upper shivplot component |
| `maxBot` | the largest y-value for the lower shivplot component |

...

### Author(s)

Owen Woody, <owoody@uwaterloo.ca>

### References

Woody, O.Z. and Nadon, R. (2006) *The Shivplot: a graphical display for trend elucidation and exploratory analysis of microarray data.* To appear in Source Code for Biology and Medicine, www.scfbm.org

### See Also

`shivplot`

---

[Package *shivplots* version 1.0 Index]
