# Supplementary material for: The Shivplot: a graphical display for trend elucidation and exploratory analysis of microarray data
Source: Source Code Biol Med. 2006 Nov 8;1:6. doi: 10.1186/1751-0473-1-6 (PMC1679802; doi:10.1186/1751-0473-1-6)
Supplement: Additional file 6 — Windows binary distribution of R shivplot package. This file is a compressed archive of the windows binary distribution of the shivplot library. [file 1751-0473-1-6-S6.addi › shivplots_1.0.zip!/shivplots/html/shiv.plotIt.html]

R: Plot a Single Shivplot on a Prepared Plotting Surface

|  |  |
| --- | --- |
| shiv.plotIt {shivplots} | R Documentation |

## Plot a Single Shivplot on a Prepared Plotting Surface

### Description

Plot a single shivplot on a previously prepared plotting surface.

### Usage

```
shiv.plotIt(data, globals, floor, plotColor, graphParams)
```

### Arguments

|  |  |
| --- | --- |
| `data` | data to be plotted |
| `globals` | axis limits, common scale parameters |
| `floor` | which position on the plotting surface this shivplot should occupy |
| `plotColor` | color to use for plotting |
| `graphParams` | additional graphing parameters |

### Details

This is an internal function of 'shivplot' - it was not meant to be called directly.

### Author(s)

Owen Woody, <owoody@uwaterloo.ca>

### References

Woody, O.Z. and Nadon, R. (2006) *The Shivplot: a graphical display for trend elucidation and exploratory analysis of microarray data.* To appear in Source Code for Biology and Medicine, www.scfbm.org

### See Also

`shivplot`

---

[Package *shivplots* version 1.0 Index]
