# Supplementary material for: The Shivplot: a graphical display for trend elucidation and exploratory analysis of microarray data
Source: Source Code Biol Med. 2006 Nov 8;1:6. doi: 10.1186/1751-0473-1-6 (PMC1679802; doi:10.1186/1751-0473-1-6)
Supplement: Additional file 6 — Windows binary distribution of R shivplot package. This file is a compressed archive of the windows binary distribution of the shivplot library. [file 1751-0473-1-6-S6.addi › shivplots_1.0.zip!/shivplots/html/shiv.plotSurface.html]

R: Prepare a Plotting Device for One or More Shivplots

|  |  |
| --- | --- |
| shiv.plotSurface {shivplots} | R Documentation |

## Prepare a Plotting Device for One or More Shivplots

### Description

Prepare a plotting device for one or more shivplots.

### Usage

```
shiv.plotSurface(numPlots, plotData, globals, plotNames, plotColors, xLab, graphParams, ...)
```

### Arguments

|  |  |
| --- | --- |
| `numPlots` | number of shivplots to be accomodated on the plotting surface |
| `plotData` | data to be plotted |
| `globals` | axis limits, common scale parameters |
| `plotNames` | names to assign to plots |
| `plotColors` | colors to use on the shivplots |
| `xLab` | label for the common x-axis shared by all present shivplots |
| `graphParams` | additional graphing parameters |
| `...` | plot surface parameters provided by the user |

### Details

This is an internal function of 'shivplot' - it was not meant to be called directly.

### Author(s)

Owen Woody, <owoody@uwaterloo.ca>

### References

Woody, O.Z. and Nadon, R. (2006) *The Shivplot: a graphical display for trend elucidation and exploratory analysis of microarray data.* To appear in Source Code for Biology and Medicine, www.scfbm.org

### See Also

`shivplot`

---

[Package *shivplots* version 1.0 Index]
