# Supplementary material for: The Shivplot: a graphical display for trend elucidation and exploratory analysis of microarray data
Source: Source Code Biol Med. 2006 Nov 8;1:6. doi: 10.1186/1751-0473-1-6 (PMC1679802; doi:10.1186/1751-0473-1-6)
Supplement: Additional file 6 — Windows binary distribution of R shivplot package. This file is a compressed archive of the windows binary distribution of the shivplot library. [file 1751-0473-1-6-S6.addi › shivplots_1.0.zip!/shivplots/html/shiv.prepGraphData.html]

R: Perform Computations Necessary to Get Points to Plot in Upper and Lower Components of the Shivplot

|  |  |
| --- | --- |
| shiv.prepGraphData {shivplots} | R Documentation |

## Perform Computations Necessary to Get Points to Plot in Upper and Lower Components of the Shivplot

### Description

Perform computations necessary to get points to plot in upper and lower components of the shivplot.

### Usage

```
shiv.prepGraphData(datax, FUN1, FUN2, LOCATION.FCN)
```

### Arguments

|  |  |
| --- | --- |
| `datax` | data to be plotted |
| `FUN1` | function for component 1 (upper half of plot) |
| `FUN2` | function for component 1 (lower half of plot) |
| `LOCATION.FCN` | function for measure of centre to use (i.e. mean) |

### Details

This is an internal function of 'shivplot' - it was not meant to be called directly.

### Author(s)

Owen Woody, <owoody@uwaterloo.ca>

### References

Woody, O.Z. and Nadon, R. (2006) *The Shivplot: a graphical display for trend elucidation and exploratory analysis of microarray data.* To appear in Source Code for Biology and Medicine, www.scfbm.org

### See Also

`shivplot`

---

[Package *shivplots* version 1.0 Index]
