# Supplementary material for: The Shivplot: a graphical display for trend elucidation and exploratory analysis of microarray data
Source: Source Code Biol Med. 2006 Nov 8;1:6. doi: 10.1186/1751-0473-1-6 (PMC1679802; doi:10.1186/1751-0473-1-6)
Supplement: Additional file 6 — Windows binary distribution of R shivplot package. This file is a compressed archive of the windows binary distribution of the shivplot library. [file 1751-0473-1-6-S6.addi › shivplots_1.0.zip!/shivplots/html/shivplots-package.html]

R: This package contains functions to create shivplots.

|  |  |
| --- | --- |
| shivplots-package {shivplots} | R Documentation |

## This package contains functions to create shivplots.

### Description

Code to produce both the standard "shivplot" (figures 1-5 from the manuscript) and the "multi-sample shivplot" (figures 6-9 from the manuscript)
is included.

### Details

|  |  |
| --- | --- |
| Package: | shivplots |
| Type: | Package |
| Version: | 1.0 |
| Date: | 2006-10-30 |
| License: | LPGL v2.1 or later |

The two most important functions in this package are 'shivplot' and 'multishivplot'. These functions generate
plots as described in the manuscript cited below. The shivplot is a graphical technique motivated by microarrays but applicable to any replicated
high-throughput data set. The plot capitalizes on the strengths of three well-established plotting
graphics (a boxplot, a distribution density plot, and a variability vs intensity plot) by effectively combining
them into a single representation.

### Author(s)

Maintainer: Owen Woody, <owoody@uwaterloo.ca>

### References

Woody, O.Z. and Nadon, R. (2006) *The Shivplot: a graphical display for trend elucidation and exploratory analysis of microarray data.* To appear in Source Code for Biology and Medicine, www.scfbm.org

### Examples

```
#not run:
## Not run: 
#Sample Commands:
shivSample(outputD=\"D://\")#Produce a series of example plots
#If a path is provided, graphical output is saved to files.
#Note:  edit(shivSample) to see how the figures are produced.
#---------
shivplot(
data=matrix(rnorm(300, 0, 1), ncol=3),
plotNames=\"Silly Test\",
plotColors=13)      #Build a shivplot out of a 100x3 random data set
                    #(100 criteria, 3 replicate observations)
#---------
exportAll(outputD=\"D://\") #Create plots from the manuscript
                            #using simulated data, output to D://

## End(Not run)
```

---

[Package *shivplots* version 1.0 Index]
