# Supplementary material for: The Shivplot: a graphical display for trend elucidation and exploratory analysis of microarray data
Source: Source Code Biol Med. 2006 Nov 8;1:6. doi: 10.1186/1751-0473-1-6 (PMC1679802; doi:10.1186/1751-0473-1-6)
Supplement: Additional file 6 — Windows binary distribution of R shivplot package. This file is a compressed archive of the windows binary distribution of the shivplot library. [file 1751-0473-1-6-S6.addi › shivplots_1.0.zip!/shivplots/html/shivSample.html]

R: Shivplot Example

|  |  |
| --- | --- |
| shivSample {shivplots} | R Documentation |

## Shivplot Example

### Description

A series of plots first showing the plots which motivated the creation of the shivplot,
and then their shivplot equivalent. Intended to help introduce the shivplot
and provide code examples (edit this function to see how things were done).

### Usage

```
shivSample(outputD = NULL)
```

### Arguments

|  |  |
| --- | --- |
| `outputD` | An output directory. If provided, graphs are exported to this location. |

### Author(s)

Owen Woody, <owoody@uwaterloo.ca>

### References

Woody, O.Z. and Nadon, R. (2006) *The Shivplot: a graphical display for trend elucidation and exploratory analysis of microarray data.* To appear in Source Code for Biology and Medicine, www.scfbm.org

### See Also

`shivplot`, `multishivplot`

---

[Package *shivplots* version 1.0 Index]
