# Supplementary material for: The Shivplot: a graphical display for trend elucidation and exploratory analysis of microarray data
Source: Source Code Biol Med. 2006 Nov 8;1:6. doi: 10.1186/1751-0473-1-6 (PMC1679802; doi:10.1186/1751-0473-1-6)
Supplement: Additional file 6 — Windows binary distribution of R shivplot package. This file is a compressed archive of the windows binary distribution of the shivplot library. [file 1751-0473-1-6-S6.addi › shivplots_1.0.zip!/shivplots/html/stdev.html]

R: Standard Deviation compatibility function

|  |  |
| --- | --- |
| stdev {shivplots} | R Documentation |

## Standard Deviation compatibility function

### Description

Allows both the R and S-PLUS function name for "standard deviation" to be used interchangeably.

### Usage

```
stdev(x, na.rm = FALSE)
```

### Details

equivalent to R's 'sd'.

### See Also

`sd`

---

[Package *shivplots* version 1.0 Index]
